# Home-Based Digital Exercise Program for Patients After Open Repair of Acute Achilles Tendon Rupture: Noninferiority Randomized Controlled Trial

**DOI:** 10.2196/78100

**Published:** 2026-03-19

**Authors:** Hui Wang, Tianyi Wu, Jiye He, Lihua Huang, Yanhong Ma, Manrong Xu, Shengdi Lu, Jian Zou

**Affiliations:** 1 Department of Orthopedics, Shanghai Xinhua Hospital, Shanghai, China; 2 Department of Orthopedics, Shanghai Sixth People's Hospital Affiliated to Shanghai Jiao Tong University School of Medicine, 600 Yishan Rd, Xuhui District, Shanghai, 200233, China; 3 Department of Rehabilitation, Shanghai Sixth People's Hospital Affiliated to Shanghai Jiao Tong University School of Medicine, Shanghai, 200233, China; 4 Department of Endocrinology, Shanghai Sixth People's Hospital Affiliated to Shanghai Jiao Tong University School of Medicine, Shanghai, 200233, China

**Keywords:** achilles tendon rupture, digital exercise program, home-based rehabilitation, physiotherapy, cost-effectiveness

## Abstract

**Background:**

Achilles tendon rupture significantly affects patient mobility and quality of life. Postoperative rehabilitation is critical to regain ankle function, strength, and return to activity. Home-based digital rehabilitation has emerged as an accessible alternative to traditional clinic-based physiotherapy; however, high-quality evidence comparing these approaches after Achilles tendon repair remains scarce.

**Objective:**

This randomized controlled trial aimed to evaluate whether a home-based digital exercise program (DEP) is noninferior to clinic-based face-to-face physiotherapy for restoring ankle range of motion (ROM), strength, patient-reported outcomes, and cost-effectiveness following open Achilles tendon repair.

**Methods:**

Between August 2020 and June 2023, 200 adult patients (mean age 36.5, SD 5.0 years; n=135, 67.5% male) undergoing open Achilles tendon repair at 2 trauma centers were randomly assigned (1:1) to either a 12-week home-based DEP group (n=100) or traditional 12-week clinic-based physiotherapy (CP group, n=100) in Shanghai. The digital program was delivered through an online app, providing instructional videos, real-time telecommunication, daily exercise tasks, and monitoring of adherence. The clinic-based group received weekly physiotherapist-supervised sessions. The primary outcome was ankle ROM at 12 weeks postoperatively. Secondary outcomes included plantarflexion strength at 0° and 12°, heel-rise index and height, Achilles Tendon Total Rupture Score (ATRS), Victorian Institute of Sport Assessment–Achilles scores, adherence, patient satisfaction, cost-effectiveness, and adverse events assessed at 6, 12, and 24 weeks. Mixed-effects models were applied for intention-to-treat analyses, and sensitivity analyses were performed in the per-protocol population.

**Results:**

At 12 weeks postoperatively, the DEP group was noninferior to the CP group for ankle ROM: mean difference (DEP–CP=−0.08°) with a 1-sided 95% CI lower bound of −2.16°, exceeding the noninferiority margin of −5° (*P*<.001). Secondary clinical outcomes at 12 weeks showed no material between-group differences, and after Holm-Bonferroni adjustment for multiplicity across secondary endpoints, no comparison reached statistical significance. Standardized effects at 12 weeks were trivial to small (|Cohen *d*|≤0.16). Patients rated DEP higher for convenience and ease of access in unadjusted analyses, but these did not remain significant after multiplicity correction (adjusted *P*=.08). DEP significantly reduced total rehabilitation costs vs CP (CNY 59,260.77 vs 68,432.80), with economic analyses favoring DEP. Adverse events were comparable between groups (DEP: 18, 18% patients and CP: 22, 22% patients; *P*>.05). The exchange rate used in this study was 1 US $=6.90 Chinese Yuan.

**Conclusions:**

The home-based DEP following Achilles tendon repair provided comparable clinical outcomes to traditional physiotherapy while significantly improving cost-effectiveness. These findings support the broader adoption of tele-supervised digital rehabilitation as a safe and effective alternative.

## Introduction

Achilles tendon rupture is one of the most common tendon injuries of the lower extremity, typically affecting physically active adults in midlife during sports activities [1]. This injury carries a substantial burden, as it often causes sudden pain, loss of function, and prolonged disability, with a profound impact on patients’ mobility and quality of life [2]. Management of acute ruptures can be surgical (open or minimally invasive repair) or nonsurgical; however, in either case, a structured rehabilitation program is critical to optimize outcomes. Early functional rehabilitation has been shown to reduce complications and improve recovery speed compared with prolonged immobilization [1,3]. Evidence from meta-analyses indicates that rehabilitative strategies emphasizing early weight-bearing and ankle mobilization after Achilles repair lead to higher patient satisfaction and faster return to sport without increasing the risk of adverse events [3]. As a result, current best practice encourages supervised, progressive rehabilitation to restore ankle range of motion (ROM), muscle strength, and functional performance after tendon healing.

Despite advances in surgical techniques, patients often experience significant deficits for months or even years after Achilles tendon injury. Studies have found that even at 1‐2 years post surgery, many individuals exhibit persistent deficits in calf muscle strength and endurance and incomplete recovery of ankle biomechanics [2,4]. These long-term impairments underscore the importance of effective rehabilitation programs to maximize functional outcomes. Standard postoperative rehabilitation for Achilles repair usually involves in-person physiotherapy sessions focusing on incremental tendon loading, gait retraining, and exercises to improve plantarflexion strength and ankle ROM. Outcomes are typically tracked using objective measures, such as ankle ROM goniometry, isometric and isokinetic plantarflexion strength testing, and single-leg heel-rise tests, as well as patient-reported outcome scores. Common validated instruments include the Achilles Tendon Total Rupture Score (ATRS) and the Victorian Institute of Sport Assessment–Achilles (VISA-A) questionnaire, which assess symptoms and functional abilities in daily activities and sports. These metrics frequently reveal that without comprehensive rehabilitation, patients may experience persistent functional deficits and lower satisfaction [2,4]. Thus, there is a strong rationale for interventions that enhance rehabilitation adherence and effectiveness after Achilles tendon repair.

In recent years, digital health and telemedicine have opened new avenues for delivering rehabilitation outside of the traditional clinic setting. Mobile health technologies, such as smartphone apps, online platforms, and videoconferencing tools, allow patients to perform prescribed exercises at home under remote guidance. Telerehabilitation programs have grown particularly prominent, accelerated by the COVID-19 pandemic, which necessitated alternatives to face-to-face therapy. Such programs typically provide instructional exercise videos, progress tracking, and sometimes real-time feedback or virtual coaching. These home-based digital exercise solutions aim to increase accessibility and patient engagement in rehabilitation, especially for those facing barriers to frequent clinic visits. Early studies in other orthopedic populations have demonstrated the potential of telerehabilitation: after knee and hip replacements, home-based telerehabilitation has yielded recovery outcomes comparable to conventional in-person physiotherapy [5]. In a 2017 systematic review of postorthopedic surgery rehabilitation, strong evidence supported telerehabilitation as an effective alternative in total joint arthroplasty, with similar improvements in function and pain as standard care [5]. Telehealth approaches can also confer practical advantages, including convenience, reduced travel time, and greater cost-effectiveness. A recent meta-analysis reported that telerehabilitation can provide equivalent or improved health outcomes while lowering overall costs and enhancing patient satisfaction and adherence to exercise programs [6]. These findings suggest that well-designed digital rehab interventions can meet the therapeutic needs of patients without the logistical constraints of clinic-based therapy.

However, despite the growing adoption of digital rehabilitation, there remains a lack of high-quality evidence specifically for Achilles tendon rupture rehabilitation. Rehabilitation after Achilles repair poses unique challenges, balancing tendon protection with early mobilization, and it is unclear whether a purely home-based app-guided regimen can match the effectiveness of hands-on physiotherapist supervision. No large randomized controlled trials have definitively compared remote digital rehabilitation to traditional in-person rehabilitation after Achilles tendon repair. Some related research in chronic Achilles conditions is promising [7], but the postoperative scenario has distinct demands and outcome measures. Given the importance of restoring ankle ROM, plantarflexion strength, and functional performance after Achilles tendon surgery, it is crucial to determine whether at-home digital programs can deliver these results on par with standard care. This study was designed to address this gap by evaluating a 12-week home-based digital exercise program (DEP) in comparison with a conventional 12-week physiotherapist-supervised rehabilitation program. By using a noninferiority randomized controlled trial, the aim is to rigorously test whether the digital home program is not worse than clinic-based rehabilitation in improving ankle mobility and functional outcomes, while also examining differences in cost-effectiveness.

## Methods

### Study Design and Setting

We conducted a 2-center, parallel-group randomized controlled trial with a noninferiority design to compare a 12-week home-based DEP with a clinic-based physiotherapy program for patients after open repair of Achilles tendon rupture. The trial took place at Shanghai Sixth People’s Hospital and Shanghai Xinhua Hospital, 2 tertiary academic centers, from August 2020 to June 2023. Eligible patients were randomized in a 1:1 ratio to the digital or conventional rehabilitation arm. We adhered to CONSORT (Consolidated Standards of Reporting Trials) [8] guidelines for trial reporting.

### Participants

Participants were recruited on the day following their emergency admission to the 2 participating hospitals. One of the coordinators screened the eligibility of participants and explained all potential harms and benefits to eligible participants before written informed consent was obtained. The inclusion and exclusion criteria are provided in Textbox 1.

Textbox 1. **Inclusion and exclusion criteria.**

**Inclusion criteria:**

- Adults (18-65 years) who were scheduled to undergo open surgical repair for an acute, complete Achilles tendon rupture.
- Surgery was planned within 2 weeks after injury.
- Participants had to commit to the rehabilitation program, follow-up visits, and be willing to provide written informed consent.
- Access to a smartphone and basic proficiency with the app.

**Exclusion criteria:**

- Participants with a history of prior Achilles tendon rupture, chronic corticosteroid use, or uncontrolled diabetes (due to potential impaired healing), or any significant comorbidity affecting the lower limb (eg, neuropathy or severe arthritis).
- Coexisting inflammatory arthritis, neurological conditions, or other lower-limb musculoskeletal disorders.
- Uncontrolled comorbidities (eg, diabetes and heart disease) that could interfere with recovery.
- Participants unable to participate in exercise (due to other injuries or contraindications) or unable to use the smartphone app or attend follow-ups.

### Intervention

All participants received standard postoperative care in the initial phase: a posterior splint or boot with the ankle in plantar flexion for 2 weeks, followed by protected weight-bearing as tolerated in a walking boot up to 6 weeks. At approximately 2‐3 weeks postsurgery (after suture removal and when acute pain subsided), participants began the assigned intervention program.

### Home-Based DEP Group

Participants randomized to the experimental group received a 12-week exercise program delivered via the “Joymotion” (Shanghai Medmotion Medical Management Co, Ltd) smartphone app. This app provides daily interactive exercise modules with video demonstrations and audio instructions for each exercise. Participants were assigned step-by-step daily rehabilitation tasks, which they performed at home while following a Physiotherapeutic Achilles-Specific Exercise program (details in Multimedia Appendix 1). The app included features for logging completed exercises and tracking progress over time. Participants could receive feedback and motivational prompts through the app, and the system flagged any missed sessions. A licensed physiotherapist remotely monitored each participant’s adherence and performance through the app’s dashboard and supervised the progression of exercises weekly, adjusting the difficulty or adding new exercises as appropriate. The therapist and participant communicated via in-app messaging or phone or video calls if issues arose, ensuring expert oversight despite the home setting. Physiotherapists maintained a high level of supervision intensity for the DEP group. They responded to participant queries within approximately 2 hours and provided feedback after each exercise session, with additional weekly summary feedback to guide the participant’s progression. We monitored intervention fidelity using the app’s dashboard metrics, including the average response time to participant messages and the frequency of feedback provided, to ensure the digital program was delivered consistently as designed. This interactive telerehabilitation approach was designed to mirror the content of standard physiotherapy in a convenient home format.

### Clinic-Based Physiotherapist Rehabilitation (CP Group)

Patients in the control group underwent a conventional rehabilitation regimen of supervised physical therapy at participating hospitals and 2 other assigned physical therapy clinics in Shanghai. This consisted of weekly in-clinic physiotherapy sessions (Physiotherapeutic Achilles-Specific Exercise program) for 12 weeks postoperative. In each session, a physiotherapist guided the patient through a standardized Achilles tendon rehab protocol, including joint mobilization, stretching, strengthening of the gastrocsoleus, balance and proprioception exercises, and functional training. The program was progressive: early sessions emphasized a gentle ROM and isometric strength exercises, advancing to resistance exercises, tendon loading drills, and functional activities in later weeks, in line with typical Achilles rehab guidelines. Between these weekly appointments, control patients were instructed in a home exercise program to perform on their own at least 5 days per week. However, unlike the digital group, they did not receive daily interactive guidance or monitoring between visits.

We recorded any deviations from the protocol or cointerventions. Compliance was encouraged in both groups, and adherence data were collected via app logs for the digital group and attendance records for the clinic group. Both groups were managed by experienced physiotherapists. We monitored adverse events throughout the program in both groups.

### Outcomes

The primary outcome was the ankle ROM of the affected side at 12 weeks postoperatively. We defined this as the active ROM in dorsiflexion (movement from the neutral ankle position to maximum dorsiflexion) measured in degrees with a goniometer. For consistency, measurements were taken with the patient supine and knee extended (to standardize gastrocnemius tension). We also recorded plantar flexion ROM, but dorsiflexion limitation was of primary interest given its functional importance after Achilles repair. At the 12-week endpoint, a blinded assessor measured the ankle ROM of both the injured and contralateral limbs; the primary analysis focused on the between-group difference in injured-side ROM.

Secondary outcomes included functional, strength, patient-reported, and economic measures, assessed at baseline (at the beginning of the intervention) and at 6-, 12-, and 24-week post surgery:

1. Plantarflexion muscle strength: isometric plantarflexion strength of the affected calf was measured at 2 ankle positions, 0° (neutral ankle) and 12° of dorsiflexion, using a dynamometer. Strength was recorded in Newton-meters (Nm) as the peak torque the patient could generate in a 3-second maximal voluntary contraction [9]. These 2 positions were chosen to assess strength across the ankle’s range (neutral vs a dorsiflexed, more elongated tendon position). Any strength deficit compared to the contralateral side was calculated as a percentage.
2. Heel-rise index and height: we evaluated calf muscle endurance and power using the single-leg heel-rise test. Patients performed single-leg heel raises to exhaustion on both the injured and uninjured sides. The heel-rise index was calculated as the ratio of the affected limb’s performance relative to the healthy side [10]. Performance was quantified as the total work or height × repetition product achieved; specifically, we counted the number of consecutive heel raises and measured the maximum heel-rise height (in cm) achieved by the affected limb and then compared it to the contralateral limb. A heel-rise index of 100% indicates full recovery (equal performance to the uninjured side), whereas lower percentages indicate residual deficits [10]. We also reported heel-rise height (the maximum height of a single heel rise on the injured side) as a separate outcome, since it reflects calf muscle function and tendon elasticity.
3. The ATRS: a validated patient-reported outcome for Achilles tendon rupture recovery. The ATRS consists of 10 items assessing symptoms and activity limitations [11]. Each item is scored 0‐10, and total scores range from 0 to 100, with 100 indicating no symptoms or functional limitations (best outcome) and 0 indicating maximal impairment. ATRS is reliable, valid, and responsive for patients with Achilles rupture and is a common primary outcome in Achilles rehabilitation studies [11].
4. The VISA-A questionnaire: originally developed for Achilles tendinopathy, we included this 8-item instrument to gauge Achilles tendon pain and function as another patient-reported metric. VISA-A scores also range from 0 to 100 (100=asymptomatic, full function). It covers pain during activity, function in daily living, and the ability to participate in sports [12]. While VISA-A is not specific to acute rupture, we hypothesized that it would capture complementary information on tendon function and any tendinopathy-like symptoms during recovery.
5. Cost-effectiveness: it was assessed by calculating the incremental cost-effectiveness ratio (ICER) between the DEP and clinic-based physiotherapy (CP) groups. Costs included intervention-related costs, health care resource usage, and productivity losses due to absenteeism and presenteeism. Data were collected from hospital records and patient-reported logs. Cost-effectiveness analysis was conducted based on the outcomes at 12 weeks of follow-up (at the end of the intervention).

All outcomes were measured by assessors blinded to group allocation (described below in the “Randomization, Allocation, and Blinding” subheading). The primary endpoint for efficacy was at 12 weeks postoperative (end of the program), with longer-term outcomes at 24 weeks used to observe maintained effects or any late differences.

### Sample Size Calculation

This trial was powered to evaluate the noninferiority of the digital rehabilitation program compared to standard physiotherapy in terms of 12-week ankle ROM. No definitive minimal clinically important difference for ankle dorsiflexion ROM after Achilles repair has been established in the literature [13]. Based on clinical judgment and prior data on variability, we set a noninferiority margin (Δ) of 5 degrees of ankle ROM. A difference of 5° was deemed the largest loss of ROM that would be considered clinically unimportant to patients. Notably, postoperative studies have found that by 6 months after Achilles repair, side-to-side dorsiflexion differences are on average near 0° (with a SD of 3°). Thus, a 5° deficit is larger than normal variability yet small enough to be functionally negligible. This choice is also consistent with the principle that the noninferiority margin should be smaller than the expected minimal clinically important difference for the outcome. Using a 1-sided type I error of .05 (α) and power (1–β) of .80, we calculated the required sample size per group for 2 independent means in a noninferiority design and estimated approximately 50 patients per group to be needed to detect noninferiority [8,14,15]. To account for potential dropouts or loss to follow-up (we anticipated 40% attrition by 24 weeks), we aimed to recruit 85 patients in each group.

### Randomization, Allocation, and Blinding

Participants were randomized in a 1:1 ratio to either the digital rehab group or the clinic-based rehab group. An independent researcher (not involved in recruitment or assessment) generated the random allocation sequence using a computer program. We used variable block sizes of 4 and 6 to ensure concealment and balanced group sizes. The allocations were sealed in opaque, sequentially numbered envelopes. After a participant was enrolled and completed all baseline assessments, the next envelope in sequence was opened to reveal the assignment. This ensured allocation concealment until the point of assignment. Randomization was not stratified (since all participants had similar baseline characteristics) and thus was purely random within blocks.

Due to the nature of the interventions, it was not possible to blind the participants or treating physiotherapists to group assignments. Participants obviously knew whether they were using an app at home or attending in-person therapy sessions. However, we implemented measures to blind outcome assessments and analyses to reduce bias. All outcome evaluations at 6, 12, and 24 weeks were performed by a research assistant blinded to the patient’s group at the outpatient department of 2 participating hospitals. This assessor was not involved in delivering the rehabilitation to either group. Participants were instructed not to reveal details of their rehabilitation program or discuss their therapy during assessment visits. The assessor recorded the outcomes without knowledge of which intervention was received. Furthermore, the trial statistician was kept blinded to group labels when analyzing primary outcomes; groups were coded anonymously until after the primary analysis was complete. By maintaining assessor and analyst blinding, we aimed to minimize measurement and analytical bias. Participants themselves were aware of their treatment, so participant blinding was not possible, but they were informed of the importance of unbiased assessment.

### Statistical Analysis

The primary outcome was the between-group difference in ankle ROM at 12 weeks. We prespecified a noninferiority margin Δ=−5° and tested H₀: μ^DEP–CP^≤−5° vs H₁: μ^DEP–CP^>−5° using a 1-sided test at *α*=.05. Mixed-effects models with fixed effects for group, time point at 6, 12, and 24 weeks, and group × time, and a random intercept for participants were used. Analyses followed a prespecified intention-to-treat (ITT) framework as primary and a per-protocol (PP) framework as sensitivity. ITT used linear mixed-effects models that incorporated all available observations under a missing-at-random assumption and retained participants in their randomized groups regardless of adherence. PP used a complete-case approach that included only participants who completed all scheduled follow-ups. Missingness occurred only at the visit level because a missed follow-up meant all variables for that visit were absent. There was no item-level missingness within attended visits.

For secondary outcomes at Week 12, we present 2-sided 95 % CIs and *P* values from the same mixed-effects model, and Holm-Bonferroni control for multiplicity across the Week 12 clinical outcomes (familywise *α*=.05); 6 and 24 weeks are presented descriptively. For the effect size, Cohen *d* at Week 12 was used as the standardized mean difference using the pooled SD from the ITT group. Patient satisfaction and acceptability items were treated as a separate hypothesis family; their *P* values were adjusted for multiplicity using the Holm-Bonferroni procedure (familywise *α*=.05). PP analyses served as sensitivity checks.

For cost-effectiveness analysis, total costs for each participant were calculated, and the difference in mean cost between groups was evaluated. In a cost-effectiveness plane, we will compare cost vs effectiveness for the 2 strategies. If the digital program were cheaper and with no worse outcome, it would be a dominant strategy. Otherwise, an ICER (cost per unit of benefit) will be computed. Nonparametric bootstrap methods (10,000 resamples) will be used to estimate uncertainty in the ICER and to construct cost-effectiveness acceptability curves.

We aimed to minimize missing data by thorough follow-up, but any remaining missing outcome data were handled under the ITT framework. The mixed-effects models can accommodate missing values under a missing-at-random assumption by using all available data for each participant. Baseline variables and other outcomes would be used in a chained equations imputation model to predict missing values, and analyses would be repeated on imputed datasets to ensure results are robust.

Data analysis was conducted using SPSS (version 28.0; IBM Corp) and R (version 4.2.2; the R Core Team). The trial results will be reported according to the CONSORT guidelines [8], including the noninferiority margin and CIs for primary outcomes.

### Ethical Considerations

Ethical approval was obtained from the Institutional Review Board of Shanghai Sixth People’s Hospital (IRB no 2020-KY-089[K]), and this approval was also acknowledged by Shanghai Xinhua Hospital. All procedures were conducted in accordance with the Declaration of Helsinki. Written informed consent was obtained from all participants before enrollment, and participants were informed of their right to withdraw from the study at any time. All personal data were anonymized (deidentified) to ensure participant privacy and confidentiality. No financial or other compensation was provided to participants for their participation in this study.

## Results

### Participants

**Figure 1.**
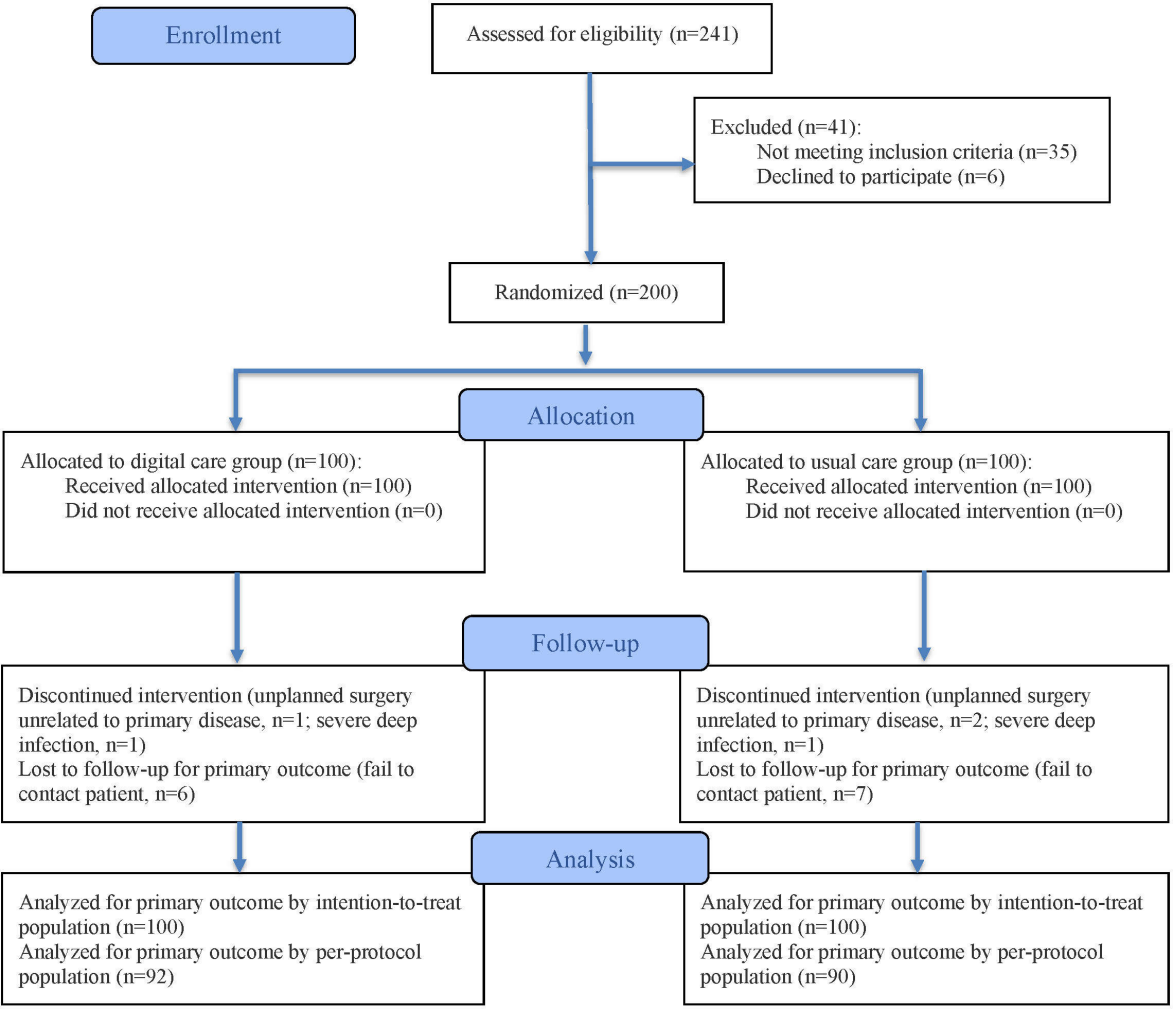
CONSORT (Consolidated Standards of Reporting Trials) flow diagram of the study.

We evaluated 241 patients with acute Achilles tendon injury, and 200 were randomized. The flowchart of our study is specified in Figure 1 (92 participants in the DEP group and 90 participants in the CP group completed the entire follow-up investigation). The baseline characteristics were comparable between the DEP and CP groups. Most participants were male, with 71 out of 100 (71%) in the DEP group and 64 out of 100 (64%) in the CP group (*P*=.21), with a mean age of approximately 37 and 36 years, respectively (*P*=.08). Average BMI was similar between groups (DEP: 25.0 kg/m^2^; CP: 25.0 kg/m^2^; *P*=.25). Education levels and insurance types were evenly distributed between the groups (*P*=.55 and *P*=.66, respectively). Smoking status did not significantly differ between groups; 17 out of 100 (17%) in the DEP group and 21 out of 100 (21%) in the CP group were smokers (*P*=.47). Injury distribution (right vs left side) was also balanced (*P*=.57). Time from rupture to surgery averaged around 3.5 days in both groups (DEP: mean 3.55, SD 0.63; CP: mean 3.59, SD 0.58; *P*=.60; Table 1).

**Table 1.** Baseline characteristics of the digital exercise program (DEP) and clinic-based physiotherapy (CP) groups.

Patient characteristic	DEP^a^ group (n=100)	CP^b^ group (n=100)	*P* value
Male patients, n (%)	71 (71.00)	64 (64.00)	.21
Age (years), mean (SD)	37 (4.93)	36 (5.14)	.08
BMI (kg/m^2^), mean (SD)	25 (0.60)	25 (0.52)	.25
Education level, n (%)			.55
Lower than high school	16 (16.00)	13 (13.00)	
Equal to or higher than high school	84 (84.00)	87 (87.00)	
Insurance type, n (%)			.66
Government	77 (77.00)	74 (74.00)	
Commercial	14 (14.00)	13 (13.00)	
Self-financed	9 (9.00)	13 (13.00)	
Current smoker, n (%)	17 (17.00)	21 (21.00)	.47
Ruptured side, n (%)			.57
Right side	55 (55.00)	52 (52.00)	
Left side	45 (45.00)	50 (50.00)	
Days from rupture to surgery, mean (SD)	3.55 (0.63)	3.59 (0.58)	.60

a DEP: digital exercise program.

b CP: clinic-based physiotherapy.

### Primary and Secondary Outcomes

The primary and secondary outcomes changes (Table 2) and mixed-effects model estimates across time (Table 3) are presented. The primary analysis supported noninferiority of DEP for ankle ROM at 12 weeks. The estimated mean difference was −0.081°(DEP – CP); the 1-sided 95% CI lower bound was −2.16°, which is above the noninferiority margin of −5° (*P*<.001). In the PP population, results were consistent (difference −0.218°; 1-sided 95% CI lower bound −2.38°; *P*<.001; Table 4).

**Table 2.** Changes in outcomes at weeks 6, 12, and 24 after surgery.

Outcome	6 weeks post surgery		12 weeks post surgery				24 weeks post surgery	
	DEP^a^ group (n=100)	CP^b^ group (n=100)	DEP group (n=100)	CP group (n=100)	Cohen *d*	Adjusted *P* value^c^	DEP^a^ group (n=100)	CP^b^ group (n=100)
Intention-to-treat population, mean (SD)								
Range of motion	16.76 (5.30)	16.40 (6.37)	31.73 (6.47)	32.73 (5.90)	—^d^	—	65.74 (6.40)	65.04 (6.03)
Plantarflexion strength 0^∘^	20.73 (3.25)	21.34 (2.90)	41.80 (3.03)	41.87 (3.31)	−0.02	1	85.02 (2.87)	85.13 (3.18)
Plantar flexion strength 12^∘^	18.47 (4.14)	18.10 (4.07)	35.95 (3.93)	36.07 (3.95)	−0.03	1	71.71 (3.83)	71.33 (4.05)
Heel-rise index	10.92 (4.53)	11.06 (3.72)	22.23 (3.69)	22.10 (3.52)	0.04	1	44.60 (4.32)	44.48 (3.81)
Heel-rise height	15.05 (4.55)	15.85 (5.47)	29.72 (5.35)	29.76 (4.74)	−0.01	1	60.51 (4.97)	61.18 (4.45)
ATRS^e^	12.06 (2.92)	11.86 (2.99)	33.33 (3.27)	32.93 (3.12)	0.13	1	53.59 (4.03)	53.75 (3.95)
VISA-A^f^	10.20 (2.85)	9.98 (3.32)	43.82 (3.78)	44.07 (4.23)	−0.06	1	65.09 (3.30)	65.63 (3.84)
Per-protocol population, mean (SD)								
Range of motion	16.71 (5.22)	16.38 (6.25)	31.54 (6.42)	32.53 (5.80)	—	—	65.93 (6.44)	64.98 (6.07)
Plantarflexion strength 0^∘^	20.63 (3.19)	21.44 (2.96)	41.75 (3.08)	41.99 (3.32)	—	—	85.04 (2.88)	85.23 (3.20)
Plantar flexion strength 12^∘^	18.35 (4.21)	18.14 (4.06)	35.92 (3.97)	35.92 (3.87)	—	—	71.68 (3.87)	71.33 (4.04)
Heel-rise index	10.92 (4.51)	10.95 (3.80)	22.34 (3.68)	22.01 (3.53)	—	—	44.52 (4.37)	44.68 (3.82)
Heel-rise height	14.96 (4.24)	15.80 (5.40)	29.48 (5.21)	29.78 (4.80)	—	—	60.62 (4.99)	61.21 (4.52)
ATRS	12.10 (2.91)	11.75 (2.86)	33.28 (3.32)	32.94 (3.11)	—	—	53.61 (3.96)	53.80 (3.98)
VISA-A	10.22 (2.91)	10.04 (3.29)	43.87 (3.81)	44.29 (4.20)	—	—	65.12 (3.20)	65.76 (3.84)

a DEP: digital exercise program.

b CP: clinic-based physiotherapy.

c Adjusted *P* value: Holm-Bonferroni adjustment.

d Not applicable.

e ATRS: Achilles tendon Total Rupture Score.

f VISA-A: Victorian Institute of Sport Assessment–Achilles.

**Table 3.** Effectiveness estimates from linear mixed-effects models.

Outcome	6 weeks post surgery		12 weeks post surgery		24 weeks post surgery	
	Coefficient (95% CI)	*P* value	Coefficient (95% CI)	*P* value	Coefficient (95% CI)	*P* value
Intention-to-treat population						
Range of motion	0.204 (−0.614 to 1.023)	.62	−0.081 (−2.563 to 2.400)	.95	0.163 (−5.254 to 5.580)	.95
Plantarflexion strength 0^∘^	−0.301 (−0.734 to 0.132)	.17	−0.039 (−3.135 to 3.058)	.98	0.03 (−5.718 to 5.777)	.99
Plantar flexion strength 12^∘^	0.214 (−0.518 to 0.947)	.56	0.205 (−2.490 to 2.899)	.88	0.325 (−4.525 to 5.174)	.90
Heel-rise index	−0.051 (−0.635 to 0.532)	.86	0.069 (−1.634 to 1.773)	.94	0.142 (−3.557 to 3.841)	.94
Heel-rise height	−0.38 (−1.083 to 0.323)	.29	−0.145 (−2.418 to 2.128)	.90	−0.209 (−5.224 to 4.806)	.94
ATRS^a^	0.117 (−0.356 to 0.59)	.62	0.289 (−2.198 to 2.775)	.82	0.231 (−3.543 to 4.006)	.90
VISA-A^b^	0.12 (−0.303 to 0.544)	.58	0.093 (−3.300 to 3.486)	.96	−0.024 (−4.828 to 4.779)	.99
Per-protocol population						
Range of motion	0.162 (−0.673 to 0.997)	.70	−0.218 (−2.789 to 2.353)	.87	0.059 (−5.639 to 5.757)	.98
Plantarflexion strength 0^∘^	−0.407 (−0.855 to 0.041)	.08	−0.329 (−3.559 to 2.901)	.84	−0.303 (−6.324 to 5.719)	.92
Plantarflexion strength 12^∘^	0.106 (−0.658 to 0.870)	.78	0.053 (−2.748 to 2.853)	.97	0.136 (−4.934 to 5.205)	.96
Heel-rise index	−0.018 (−0.626 to 0.590)	.95	0.089 (−1.690 to 1.867)	.92	0.025 (−3.866 to 3.916)	≥.99
Heel-rise height	−0.421 (−1.112 to 0.27)	.23	−0.358 (−2.716 to 1.999)	.77	−0.43 (−5.702 to 4.841)	.87
ATRS^a^	0.179 (−0.316 to 0.674)	.48	0.217 (−2.379 to 2.814)	.87	0.129 (−3.818 to 4.076)	.95
VISA-A^b^	0.091 (−0.355 to 0.537)	.70	−0.083 (−3.646 to 3.480)	.96	−0.241 (−5.267 to 4.786)	.93

a ATRS: Achilles tendon Total Rupture Score.

b VISA-A: Victorian Institute of Sport Assessment–Achilles.

**Table 4.** Primary outcome (ankle range of motion [ROM]) noninferiority analysis at 12 weeks. Difference is digital exercise program (DEP) – clinic-based physiotherapy (CP); noninferiority margin (Δ)=−5°.

Population	Mean difference (°)	One-sided 95% CI lower bound (°)	Noninferiority *P* value
Intention-to-treat population	−0.081	−2.16	<.001
Per-protocol population	−0.218	−2.38	<.001

At Week 12, unadjusted model estimates showed no significant between-group differences for plantarflexion strength (0° and 12°), heel-rise index, heel-rise height, ATRS, or VISA-A. After Holm-Bonferroni correction across these 6 secondary clinical endpoints, none was significant (all adjusted *P*≥.99).

To contextualize magnitudes, Week 12 Cohen *d* values indicated trivial to small effects: ankle ROM d=−0.16; plantarflexion strength 0° d=−0.02; plantarflexion strength 12° d=−0.03; heel-rise index d=+0.04 ; heel-rise height d=−0.01; ATRS d=+0.13; VISA-A d=−0.06 (positive d favors DEP; Table 2). We summarized participant-level and visit-level missingness by group and time point in Table S1 in MultimediaAppendices 1  2.

### Cost-Effectiveness Analysis

The average total cost per participant was significantly lower in the DEP group compared to the CP group during the 24-week postoperative period (CNY 59,260.77 vs CNY 68,432.80; difference=−9172.03; *P*<.001). The exchange rate used in this study was 1 US $=6.90 Chinese Yuan. Major cost savings for the DEP group arose from rehabilitation costs (difference=−3932.65; *P*<.001), primary care expenses (difference=−1820.62; *P*<.001), secondary care costs (difference=−2245.19; *P*<.001), medication (difference=−1073.66; *P*<.001), and transportation (difference=−960.10; *P*<.001). Incremental cost-effectiveness analysis showed negative ICER values for primary and secondary outcomes, indicating cost savings without clinically relevant differences between groups. Specifically, the ICER for ankle ROM was CNY 113,234.94 per additional unit improvement, highlighting significant cost savings without sacrificing clinical effectiveness. Similar patterns were observed for secondary outcomes, including ATRS (ICER=−31,737.13) and VISA-A (ICER=−98,623.98), reinforcing the economic advantage of the digital rehabilitation program (Tables5  6).

**Table 5.** Average total cost per patient in the digital exercise program (DEP) group and clinic-based physiotherapy (CP) group during the 24 weeks after the postoperative period.

Cost category (CNY^a^)	DEP^b^ group (n=100), mean (SD)	CP^c^ group (n=100), mean (SD)	*P* value
Intention-to-treat population			
Rehabilitation cost	6980.00 (0)	10912.65 (1509.25)	<.001
Hospital stay cost	6903.08 (962.78)	6809.41 (998.34)	.51
Primary care cost	121.13 (48.32)	1941.75 (268.55)	<.001
Secondary care cost	4886.38 (948.22)	7131.57 (2160.98)	<.001
Paid home cost	550.04 (203.51)	782.28 (282.86)	<.001
Medication	6941.91 (1416.06)	8015.57 (1588.23)	<.001
Transportation cost	518.80 (199.38)	1478.90 (268.81)	<.001
Nutrition cost	2945.16 (493.63)	3039.46 (456.52)	.17
Lost wages for patients	26383.63 (14552.00)	25727.87 (12775.52)	.74
Lost wages for families	3030.64 (3397.40)	2583.35 (3313.99)	.35
Total cost	59260.77 (15099.91)	68432.80 (13750.30)	<.001
Per-protocol population			
Rehabilitation cost	6980.00 (0)	10921.53 (1482.25)	<.001
Hospital stay cost	6943.15 (924.93)	6848.47 (990.64)	<.001
Primary care cost	120.11 (47.50)	1943.33 (263.75)	.51
Secondary care cost	4873.82 (953.44)	7009.79 (2143.54)	<.001
Paid home cost	555.10 (205.82)	783.13 (274.64)	<.001
Medication	6889.73 (1412.89)	7986.04 (1574.05)	<.001
Transportation cost	516.24 (200.37)	1476.30 (274.05)	<.001
Nutrition cost	2908.00 (474.83)	3037.04 (463.03)	<.001
Lost wages for patients	27061.77 (14540.13)	25610.86 (12444.39)	.07
Lost wages for families	3073.00 (3423.92)	2434.28 (3179.87)	.47
Total cost	59920.91 (15162.77)	68050.76 (13462.49)	.19

a CNY: Chinese YUAN.

b DEP: digital exercise program.

c CP: clinic-based physiotherapy.

**Table 6.** Incremental cost-effectiveness ratio (ICER).

Population and analysis	Incremental cost (CNY^a^)	Range of motion, mean (95% CI)	Plantarflexion strength 0^∘^, mean (95% CI)	Plantarflexion strength 12^∘^, mean (95% CI)	Heel-rise index, mean (95% CI)	Heel-rise height, mean (95% CI)	ATRS^b^, mean (95% CI)	VISA-A^c^, mean (95% CI)
Intention-to-treat population								
Main analysis (mixed effects)	−9172.03	−0.081 (−2.563 to 2.400)	−0.039 (−3.135 to 3.058)	0.205 (−2.490 to 2.899)	0.069 (−1.634 to 1.773)	−0.145 (−2.418 to 2.128)	0.289 (−2.198 to 2.775)	0.093 (−3.300 to 3.486)
ICER^d^	—	113,234.94	235,180.26	−44,741.61	−132,927.97	63,255.38	−31,737.13	−98,623.98
Per-protocol population								
Main analysis (mixed effects)	−8129.85	−0.081 (−2.563 to 2.400)	−0.039 (−3.135 to 3.058)	0.205 (−2.490 to 2.899)	0.069 (−1.634 to 1.773)	−0.145 (−2.418 to 2.128)	0.289 (−2.198 to 2.775)	0.093 (−3.300 to 3.486)
ICER	—	100,368.52	208,457.69	−39,657.80	−117,823.91	56,067.93	−28,130.97	−87,417.74

a CNY: Chinese Yuan.

b ATRS: Achilles tendon Total Rupture Score.

c VISA-A: Victorian Institute of Sport Assessment–Achilles.

d ICER: incremental cost-effectiveness ratio.

### Patients’ Adherence

In the DEP group, participants completed a mean of 28.5 (SD 2.6) of 30 educational modules and attended 5.7 (SD 1.1) of 6 scheduled video consultations (metrics specific to the digital program; not applicable to the CP group). At 24 weeks, patient ratings were high in both groups. Although mean scores favored DEP for convenience (mean 4.6, SD 0.5 vs mean 4.4, SD 0.7) and ease of use and access (mean 4.4, SD 0.7 vs mean 4.2, SD 0.5), these differences were not significant after Holm-Bonferroni adjustment within the satisfaction or acceptability family (both adjusted *P*=.08). Helpfulness (mean 4.7, SD 0.4 vs mean 4.7, SD 0.5; adjusted *P*≥.99) and communication with the physiotherapist (mean 4.8, SD 0.4 vs mean 4.7, SD 0.4; adjusted *P*=.16) were similarly high and comparable between groups (Table 7).

**Table 7.** Patients’ adherence to treatment (intention-to-treat population).

Outcome measure	DEP^a^ group (n=100), mean (SD)	CP^b^ group (n=100), mean (SD)	Adjusted *P* value
Educational modules completed (maximum of 30 modules)	28.5 (2.6)	N/A^c^	—
Number of video consultations attended (maximum of 6 times)	5.7 (1.1)	N/A	—
Patients’ scoring for intervention at 24 weeks			
Convenience (1-5)	4.6 (0.5)	4.4 (0.7)	.08
Ease of use and access (1-5)	4.4 (0.7)	4.2 (0.5)	.08
Helpfulness (1-5)	4.7 (0.4)	4.7 (0.5)	≥.99
Communication with the physiotherapist (1-5)	4.8 (0.4)	4.7 (0.4)	.16

a DEP: digital exercise program.

b CP: clinic-based physiotherapy.

c N/A: not applicable.

### Adverse Events

The incidence of adverse events was similar between the DEP and CP groups: 18 out of 100 (18%) patients in the DEP group vs 22 out of 100 (22%) patients in the CP group experienced an adverse event. Most adverse events were related to therapy, primarily ankle pain (DEP: 8 and CP: 10) and swelling (DEP: 6 and CP: 5). Muscle strain occurred equally (2 per group). Serious adverse events were rare, occurring in 3 out of 100 (3%) patients in each group, including deep infections (DEP: 1 and CP: 1) and unrelated surgeries (DEP: 1 and CP: 2). One patient in the DEP group withdrew because of relocation. No substantial differences in safety profiles emerged, suggesting comparable tolerability and safety of both rehabilitation programs (Table 8).

**Table 8.** Adverse events and serious adverse events.

Adverse events	DEP^a^ group (n=100)	CP^b^ group (n=100)
Patients with adverse events, n (%)	18 (18)	22 (22)
Events related to study therapy, n (%)	17 (17)	17 (17)
Events unrelated to study therapy, n (%)	4 (4)	8 (8)
Type of event, n (%)		
Involved ankle		
Pain	8 (8)	10 (10)
Swelling	6 (6)	5 (5)
Muscle strain^c^	2 (2)	2 (2)
Signs of infection^d^ (swelling, redness, heat, or pus)	1 (1)	2 (2)
Mobilization under anesthesia	1 (1)	0 (0)
Other		
Nausea and dizziness	0 (0)	2 (2)
Insomnia	1 (1)	1 (1)
Anxiety about ankle recovery	2 (2)	3 (3)
Serious adverse events^e^, n (%)		
Patients with serious adverse events	3 (3)	3 (3)
Events related to study therapy	1 (1)	1 (1)
Events unrelated to study therapy	2 (2)	2 (2)
Unplanned surgery, which is unrelated to primary injury	1 (1)	2 (2)
Moving to another city	1 (1)	0 (0)
Deep infection	1 (1)	1 (1)

a DEP: digital exercise program.

b CP: clinic-based physiotherapy.

c Four patients sustained gastrocnemius muscle strain during exercise with minor consequent symptoms.

d Three patients developed superficial infection and eventually healed with conservative treatment.

e Patients with serious adverse events were automatically withdrawn from the study.

## Discussion

### Principal Findings

This randomized controlled trial demonstrated that the home-based DEP was noninferior to traditional clinic-based physiotherapy for patients following open Achilles tendon repair. At the primary endpoint, ankle ROM showed no significant differences between groups, affirming the effectiveness of the digital rehabilitation approach. Similarly, no meaningful differences emerged across secondary outcomes, including plantarflexion strength, heel-rise tests, and patient-reported outcomes, at all time points assessed (6, 12, and 24 weeks). Importantly, the DEP strategy achieved these clinical results at significantly lower costs, with average savings of approximately CNY 9172 per patient compared to the conventional approach. Incremental cost-effectiveness analyses further indicated that DEP delivered equivalent or improved cost-effectiveness across all measured outcomes. Additionally, adherence to rehabilitation was notably high in the digital group, where participants reported greater convenience and ease of use compared to face-to-face rehabilitation. Adverse events and serious adverse events were infrequent and evenly distributed between the 2 groups, suggesting comparable safety profiles. Collectively, these findings suggest that a structured digital home rehabilitation program can effectively substitute clinic-based physiotherapy after Achilles tendon repair, offering a safe, cost-effective, and patient-friendly alternative.

Our noninferiority analysis using a 1-sided 95% CI lower bound confirmed that DEP is not inferior to CP for restoring ankle ROM at 12 weeks. Importantly, standardized effect sizes were uniformly small (|d|≤0.16), emphasizing that between-group differences were not only statistically nonsignificant after multiplicity control but also clinically negligible in magnitude. Furthermore, multiple-adjusted analyses of secondary endpoints showed no clinically relevant advantages for CP over DEP. While unadjusted patient-reported convenience and access ratings favored DEP, these did not meet family-wise significance after correction and should be interpreted as exploratory.

A high-quality randomized controlled trial on acute Achilles tendon rupture rehabilitation found that a home-based digital rehabilitation program produced clinical outcomes equivalent to conventional in-clinic physiotherapy. Patients in the digital rehab group achieved similar ankle ROM, calf muscle strength, and patient-reported outcome measures as those attending standard physiotherapy, indicating noninferiority of the telehealth approach. This finding is consistent with prior evidence in related conditions, for example, a noninferiority trial in chronic Achilles tendinopathy reported no worse pain or functional outcomes with telehealth-only or hybrid care compared to all in-person treatment [7]. Such results suggest that when rehab protocols are well-designed and supervised remotely, patients can attain comparable functional recovery to traditional face-to-face therapy.

Our trial’s results align with broader Achilles rupture rehabilitation evidence, emphasizing that rehabilitation protocols, rather than the setting, drive outcomes. Past research shows that early functional rehabilitation is critical for optimal recovery after Achilles repair. For example, whether managed operatively or nonoperatively, patients who undergo an accelerated rehab program (early weight-bearing and mobilization) attain excellent strength, ROM, and return-to-activity outcomes [16]. Willits et al [16] demonstrated that nonoperative treatment with an aggressive rehab protocol yielded strength and functional scores equivalent to surgical repair, highlighting rehab’s importance in tendon healing. In practice, many components of Achilles rehab (joint mobilizations, progressive loading exercises, and balance training) can be instructed and progressed remotely. A recent small study even found that an unsupervised, home-based exercise program after Achilles repair achieved high VISA-A scores and no increase in complications compared to traditional supervised therapy [17]. These findings reinforce that, as long as evidence-based rehabilitation principles are followed, including protection in early healing and progressive loading to restore tendon capacity, outcomes after Achilles rupture can be excellent with either clinic or home-based guidance. The digital model simply provides a different delivery mechanism for the same key interventions, and the trial confirms it can do so without compromising efficacy.

Growing evidence in other musculoskeletal conditions bolsters the trial’s implications. In total joint arthroplasty, multiple studies have shown telerehabilitation to be comparable to standard outpatient rehabilitation for restoring function. A comprehensive meta-analysis of 11 randomized controlled trials in posttotal knee replacement patients found no significant differences in pain reduction or functional improvement between telehealth and in-person rehabilitation, with telerehabilitation yielding similar gains in walking ability, strength, and ROM [18]. Notably, remote programs often come with lower health care usage and cost. Tsang et al [18] reported that telerehabilitation after knee replacement significantly reduced hospital visits and was more cost-effective than usual care. Likewise, technology-assisted rehabilitation after hip or knee arthroplasty has been shown to improve pain and mobility outcomes on par with or better than usual care, partly by enabling more frequent exercise and immediate feedback through apps or video coaching [19]. Beyond postsurgical care, tele-physiotherapy has proven effective in chronic orthopedic conditions. For instance, in knee and hip osteoarthritis and tendinopathies, real-time video- or phone-based rehabilitation produces pain relief and functional gains equivalent to face-to-face therapy [7,19]. Systematic reviews consistently conclude that telerehabilitation is a safe and effective modality for musculoskeletal rehabilitation, with outcomes in pain, strength, and quality of life similar to traditional care across diverse conditions [7,19].

Importantly, some studies suggested that telehealth models can confer additional advantages, such as greater exercise adherence and empowerment. Patients often become more actively involved in self-management when guided remotely, which can enhance long-term exercise habits. However, clinicians implementing telerehabilitation should ensure proper patient selection and education. Key success factors include an initial in-person assessment when needed, clear exercise instructions (via videos or mobile apps), and regular follow-up to maintain accountability. When these conditions are met, the literature strongly supports that digital rehabilitation matches conventional physiotherapy in outcomes for Achilles tendon repair and beyond, offering a convenient alternative without loss of efficacy [18,19].

Interactive telerehabilitation may improve exercise adherence. In a randomized trial after total knee arthroplasty, patients using a guided telehealth platform had significantly higher adherence to prescribed exercises than the usual-care group [20]. The engaging digital interface and at-home convenience can motivate patients to complete rehabilitation exercises regularly.

Overall, patient satisfaction with telerehabilitation is high when implemented properly. During the COVID-19 pandemic, both patients and physiotherapists reported positive experiences with telehealth musculoskeletal care [21]. Patients often appreciate the flexibility and reduced travel burden, and many find remote coaching effective for guidance and feedback. That said, some individuals may miss hands-on contact; addressing patient education and ensuring a user-friendly technology experience are important for maintaining satisfaction.

### Clinical Implication of the Study

Our cost-effectiveness analysis confirmed that the DEP intervention was a dominant strategy, providing equivalent effectiveness at a significantly lower cost compared to the clinic-based physiotherapy program. This study has significant clinical implications, indicating that home-based digital rehabilitation can effectively substitute traditional in-clinic physiotherapy following Achilles tendon repair. Clinicians can confidently implement structured telerehabilitation programs, which provide comparable clinical outcomes with additional benefits such as cost savings, convenience, and high patient adherence. Particularly, patients with geographic, logistical, or time constraints may benefit substantially from remotely supervised rehabilitation. Health care systems could leverage digital rehabilitation solutions to enhance resource allocation efficiency and reduce health care costs without compromising clinical quality. Moreover, physiotherapists can expand their service delivery through digital platforms, improving patient engagement, continuity of care, and accessibility to expert guidance. However, careful patient selection and initial training remain essential to optimize telehealth outcomes, underscoring the necessity for ongoing professional education in digital rehabilitation practices.

### Strength and Limitation

This study’s strengths include its rigorous randomized controlled trial design, adequate sample size based on a clear noninferiority margin, and comprehensive assessment of both clinical and economic outcomes. Adherence and satisfaction were meticulously monitored, enhancing practical insights into patient acceptability of digital rehabilitation. However, limitations must be acknowledged. First, blinding participants and therapists was inherently impossible, potentially introducing performance bias. Second, follow-up was relatively short; thus, longer-term outcomes remain uncertain. Third, generalizability may be restricted, as the study design focused predominantly on younger, technology-proficient patients. Fourth, the use of the VISA-A score, a scale originally validated for chronic tendinopathy, has inherent limitations in acute rupture patients [13,22]. Some VISA-A items (eg, 30-minute walking endurance) are not feasible in early postoperative rehabilitation, making the score insensitive to early improvements [13,22]. Moreover, VISA-A changes do not necessarily reflect structural tendon healing, and the instrument is generally less responsive to recovery progress in acute ruptures compared to the ATRS [11]. We included the VISA-A as a complementary patient-reported measure of tendon-related symptoms, but we interpret its findings alongside objective outcomes (ankle ROM and calf strength) for a more comprehensive assessment [11]. Given our cohort’s relatively young mean age and requirement for smartphone use, older adults and those with limited digital literacy were underrepresented. These groups often face barriers to telerehabilitation (eg, limited internet access and difficulty using smartphone interfaces), so the generalizability of DEP to these populations is uncertain. Finally, despite rigorous adherence monitoring, actual exercise intensity and fidelity to prescribed techniques in home settings were not objectively quantified, possibly influencing rehabilitation outcomes.

### Conclusion

Home-based digital exercise after Achilles tendon repair was clinically noninferior to traditional clinic-based physiotherapy, providing similar functional outcomes, safety, greater patient convenience, adherence, and significant cost savings. These findings support broader adoption of structured telerehabilitation programs, offering effective, accessible, and patient-centered postoperative care. Further research should evaluate long-term outcomes and applicability across diverse patient populations.

## Supplementary material

10.2196/78100 Multimedia Appendix 1 Physiotherapeutic Achilles-Specific Exercise (PASE) program aligned with early accelerated rehabilitation protocols.

10.2196/78100 Multimedia Appendix 2 Follow-up attendance by group and scheduled visit.

10.2196/78100 Checklist 1 CONSORT-eHEALTH checklist (version 1.6.1).

## Abbreviations

ATRS: Achilles Tendon Total Rupture Score
CONSORT: Consolidated Standards of Reporting Trials
CP: clinic-based physiotherapy
DEP: digital exercise program
ICER: incremental cost-effectiveness ratio
ITT: intention-to-treat
PP: per-protocol
ROM: range of motion
VISA-A: Victorian Institute of Sport Assessment–Achilles
