# Supplementary material for: Home-Based Digital Exercise Program for Patients After Open Repair of Acute Achilles Tendon Rupture: Noninferiority Randomized Controlled Trial
Source: JMIR Mhealth Uhealth. 2026 Mar 19;14:e78100. doi: 10.2196/78100 (PMC13002159; doi:10.2196/78100)
Supplement: Multimedia Appendix 1 [file mhealth-v14-e78100-s001.docx]

**Supplemental Materials**

*Intervention*

Physiotherapeutic Achilles-Specific Exercise (PASE) program aligned with early accelerated rehabilitation protocols^1-9^

| **Week** | **Status & Key Assessments** | **Rehabilitation Content** | **Home Exercises** | **Adherence & Adjustments** |
| --- | --- | --- | --- | --- |
| **Week 1** (Post-op Days 0–7) | Status: NWB in splint or cast, ankle ~20–30° plantarflexion. Wound intact with dressings.  Assess: Check surgical wound (no redness or drainage), signs of DVT, pain level, neurovascular status of foot/toes. Ensure patient understands NWB precautions. | - Goal: Protect repair, allow early healing of tendon and incision. Mobilization: *Ankle:* None (immobilized in equinus). *Weight-Bearing:* NWB – use crutches or knee scooter; foot elevated when resting. - Therapy Sessions: No in-person visits; 1 remote orientation call to reinforce instructions (e.g. proper crutch use, app setup). - Adjacent Joints: Begin gentle active motion of toes, knee, and hip on the injured leg to prevent stiffness (e.g. knee bends, hip flex/ext). Unaffected leg and upper body can do light exercise. - Pain Management: Elevate leg above heart and apply ice (around cast) 15–20 min, 3–4×/day to reduce edema. Take analgesics as prescribed. Avoid disturbing the wound (keep dry). - Manual Therapy: None on Achilles. Emphasis on rest. (Patient may do gentle ankle pumps in cast only if cleared, primarily to encourage circulation, not true motion at repair site.) | - Foot/toe mobility: Toe curls (spread and scrunch toes inside cast); 10 reps, 3×/day. - Quadriceps & Glute Sets: Isometric thigh squeezes and buttock squeezes; 10 reps, 3×/day. - Straight Leg Raise: Supine, lift injured leg (knee straight) to engage thigh; 10 reps, 2×/day (if tolerable). - Upper Body/Cardio: Seated arm exercises (light weights or bands) or gentle conditioning (to maintain fitness), as comfort allows. | - Adherence: Strict NWB – use assistive device at all times (no weight on foot). Log daily pain, swelling, and exercise completion in app. Maintain elevation ~90% of time to minimize swelling. - Adjustments: If pain or swelling is excessive, consult the surgeon for adjust pain meds. If cast causes pressure or numbness, seek medical advice. Reinforce that smoking is to be avoided (impairs tendon healing). Patients read educational materials on tendon repair and rehab timeline to set expectations. |
| **Week 2** | - Status: ~1–2 weeks post-op. Likely first post-op visit: sutures/staples removed if wound healed. Transition from splint to CAM boot (set in plantarflexion with heel wedges). - Assess: Incision status (dry, closed), calf muscle tone (expect inhibition), ankle resting position, and patient’s ability to perform gentle toe movement. Screen for calf tenderness or Homan’s sign (DVT). Review patient’s pain levels and app log from week 1. | - Goal: Initiate *protected* motion and minor loading without compromising repair. - Mobilization: *Ankle:* May begin active plantarflexion in boot or with foot out of boot if allowed, but limit dorsiflexion to neutral (0°) – do not stretch Achilles. *Weight-Bearing:* Begin partial WB (~10–20% body weight) in boot *if* surgeon approves. Patients use two crutches, touching foot lightly down for balance. - Sessions: 1 tele-rehab session to teach safe partial weight-bearing technique and initial ankle exercises. Verify proper boot fit (with ~2–3 cm heel lift) and crutch use. - Adjacent Joints: Continue knee and hip ROM (e.g. gentle knee flexion/extensions, hip abduction). Add light resistance for quads/hamstrings if possible (e.g. quad sets with rolled towel). Maintain mobility in toes (wiggling) to prevent stiffness. - Pain Mgmt: Pain should be decreasing. Continue ice/elevation after exercises. If needed, use NSAIDs (if approved) for pain/swelling control, but avoid overuse to not mask pain as a guide. - Manual Therapy: Scar – if incision is fully closed and dry, patient can perform gentle scar mobilization around wound edges (two fingers lightly massaging in circles) 1–2×/day to prevent adhesions. No direct deep massage on tendon. No joint mobilizations yet (ankle is still protected). | - AROM Initiation: Ankle pumps (active PF to neutral): With boot off for exercise, gently point foot down and back *to neutral only*; 10–15 reps, 3×/day. (Keep knee bent to reduce stretch on tendon.) - Isometric PF: With leg in boot or supported, gently press foot down (as if pressing gas pedal) without moving ankle (submaximal effort); hold 5 sec, 10 reps, 3×/day. (No pain.) - Toe curls & towel scrunch: Spread toes then curl them or scrunch a towel on the floor (if out of boot for exercises); 10 reps, 2–3×/day. - Hip/Knee strength: Straight-leg raises (as in week 1) and side-lying hip abductions (lift injured leg sideways); 10 reps, 2×/day each. Continue core/upper-body conditioning (non-weightbearing). | - Adherence: Wear boot *at all times* except for hygiene and exercises. Keep the app log – record each exercise session and pain level (expect discomfort <3/10 with motion). Use a bathroom scale to gauge partial weight (practice weight-shifting to ~20% body weight). - Adjustments: If active motion causes sharp pain or pulls at incision, back off range and inform PT/surgeon. If wound shows any redness or oozing after exercises, pause movement and contact provider. Ensure patient doesn’t *overdo* weight-bearing – emphasize that strict partial WB is critical (early loading is beneficial but must be controlled). Encourage questions via app messaging to address any uncertainty. |
| **Week 3** | - Status: 2–3 weeks post-op. Patient in boot with wedges, beginning to increase WB. Incision healing well (scar forming). - Assess: Ankle resting angle in prone (with knee 90°) to monitor tendon length – should still be slightly plantarflexed relative to uninjured side (no excessive dorsiflexion). Gently assess active plantarflexion strength (ability to press foot down against minimal resistance). Evaluate gait quality with partial WB and crutches (is patient able to bear ~25–50% weight without pain?). Check for swelling (around ankle or foot) after increased activity. | - Goal: Safely progress weight-bearing and maintain ankle mobility without elongating tendon. - Mobilization: *Ankle:* Continue active ROM exercises in pain-free range (plantarflexion and slight dorsiflexion, not beyond neutral). May gradually increase dorsiflexion *toward* neutral as tolerated (still avoid any stretch). *Weight-Bearing:* Increase to ~50% PWB in boot as tolerated – patient can start using one crutch or cane on opposite side for short distances, while still using two crutches for longer distances. - Sessions: Remote check-in (video) to observe partial weight-bearing gait and ensure correct technique (shorter stride to reduce tendon strain). Adjust exercises if needed based on pain/effort. - Adjacent Joints: Add gentle ankle inversion/eversion AROM (with boot off, move foot inward and outward slightly) to maintain subtalar mobility – ensure motions are within protected range (no sharp pain). Continue knee ROM and start gentle hamstring stretches (towel behind thigh) if hamstring is tightening from altered gait. - Pain Mgmt: Likely off strong pain meds; use ice after rehab exercises if mild swelling. Compression sock or light ACE wrap on lower leg may be used during day to control edema (ensure no pressure on incision). - Manual Therapy: Continue self-scar massage (scar mobilization) along the healing incision and surrounding tissue to prevent adhesions. Soft tissue: patient can do light self-massage to calf muscle (gastroc-soleus) *above* the repair site to reduce muscle tightness, but avoid direct pressure on tendon. No passive Achilles stretching. | - WB Practice: Weight-shift drills: In standing (boot on), shift weight gently forward/back and side-to-side, keeping within 50% WB limit; 2–3 minutes, 3–4×/day. - Active ankle ROM: Continue ankle pumps to neutral and - alphabet exercises (trace the alphabet in the air with foot) within safe range; 10 min total ROM work per day (split into sessions). - TheraBand light PF: If approved, start band plantarflexion (light resistance band around ball of foot, push down); 2 sets of 10, 1–2×/day. *Only go to neutral, not into strong stretch.* - Stationary cycling (gentle): If available, bike with boot on, low resistance, for 10 min to promote circulation (incision must be healed); 1×/day. - Hip/Core: Continue hip abductions, add bridging (bend knees, lift hips) – both legs support; 10 reps, 2×/day. | - Adherence: Patient should use the app to track *weight-bearing progression* – e.g. note number of steps or time spent standing/walking each day, aiming for gradual increases. Continue using one crutch as needed; do not limp without support. - Adjustments: If patient reports a feeling of “excessive looseness” in the tendon or observes foot hanging more dorsiflexed, pause progression and inform provider (monitoring for tendon elongation). If partial WB is pain-free and stable, therapist may allow slightly faster progression; if painful, hold at current level longer. Reinforce confidence with weight-bearing – explain that early loading (within limits) *will not harm the repair* and actually improves outcomes. |
| **Week 4** | - Status: 3–4 weeks post-op. Boot still on, possibly with one wedge removed to gradually decrease plantarflexion (ankle angle moving closer to neutral). Patient likely ~75% weight-bearing in boot with minimal pain. - Assess: Gait in boot – is the patient walking with nearly normal stance duration on injured side (with crutch support)? Inspect calf for muscle atrophy (notable but activation should be improving). Measure active dorsiflexion (knee bent) – should approach neutral (0°) but not beyond. Confirm no signs of tendon gap or excessive elongation (Thompson test should show some foot movement; resting ankle angle still slightly plantarflexed relative to contralateral). Check scar maturity and any adhesions (scar should be maturing; continue to be mobile). | - Goal: Achieve full weight-bearing (FWB) in boot by ~4–6 weeks mark; continue to improve ankle ROM to neutral and gentle strength. - Mobilization: *Ankle:* Begin *active* dorsiflexion toward neutral with a bit more force (still no passive stretch). If a wedge was removed, ankle is less plantarflexed now – monitor tolerance. *Weight-Bearing:* Progress to FWB in boot as tolerated. By end of week 4, many patients can stand and walk short distances in the boot without crutches, using a normal gait pattern (heel strike on boot rocker). Encourage increasing weight on injured leg during daily activities, using crutch only if limp or discomfort. - Sessions: Telehealth video session to evaluate FWB gait. Therapist checks that patient can walk in boot with symmetry (no toe-walking on that side, stride length even) and gives cues to normalize gait. Discuss transitioning off crutches completely when gait is without a significant limp. - Adjacent Joints: Introduce gentle toe extension stretch (manual big-toe dorsiflexion) to ensure toe mobility (boot often keeps toes still). Continue full knee extension/flexion exercises (e.g. mini-squats within pain-free range, see below). - Pain Mgmt: By now pain is usually low. Emphasize post-activity icing if swelling occurs. Night: may remove boot *briefly* for comfort with ankle supported in slight PF (if surgeon allows) – this can improve sleep, but patient must put boot on before standing. - Manual Therapy: If scar tissue is adhering (skin stuck to underlying tissue), teach more aggressive scar massage (using lotion, two fingers to mobilize scar up/down and in circles for several minutes). Joint mobilizations: Patient can perform self-mobilization for ankle joint in protected range – e.g. gentle anterior-posterior glide by pulling foot up with a strap while in slight plantarflexion (not past neutral). These should be very gentle. Still no passive Achilles stretch until later. | - Strength: Seated calf raises (double-leg): Sit with feet on floor (boot on for injured side), gently push up through toes lifting heels off ground – most weight will be on uninjured side, but injured side starts to take some load; 2 sets of 15, 1–2×/day. *(This begins closed-chain loading in a protected way.)* - Theraband exercises: Increase resistance band level if PF is pain-free – 3 sets of 10 plantarflexion, 1×/day. Also add inversion/eversion with band (light resistance, 2×10 each) to keep ankle and subtalar joint mobile and the supporting muscles (tibialis posterior, peroneals) active. - Mini-squats: With boot on and holding a support, perform shallow squats (knee bend 0–45°) keeping weight equal on both legs; 2 sets of 10, 1×/day. (Avoid going so deep that ankles dorsiflex beyond neutral). - Stationary bike: If available, increase to 15–20 min as tolerated (still with boot or brace on foot). - Balance: Begin tandem stance (one foot in front of the other) or semi-tandem stance, eyes open, to challenge balance gently; 30 sec hold, 3×. | - Adherence: By this phase, the patient should be walking in the boot most of the day. Emphasize full-time boot wear until criteria met (usually ~6–8 weeks for boot wean). The app can send daily reminders to do walking practice and log step counts. Patient should aim to increase activity volume *gradually* – e.g. +5 minutes of walking in boot each day if no increase in pain/swelling. - Adjustments: If gait is still very antalgic or patient is apprehensive to bear weight, schedule an extra tele-session for coaching. Some patients may progress slower; if FWB is not reached by end of week 4, continue gradual increase – better to reach FWB by week 5 than risk a setback. If persistent ankle stiffness is noted (can’t reach neutral), therapist may hold off removing further wedges until week 5 and encourage more active ROM exercise. Positive reinforcement is key – remind patient that early weight-bearing and motion improve outcomes (earlier return of function, high satisfaction) without raising complication rates. |
| **Week 5** | - Status: 4–5 weeks post-op. Patient should be FWB in boot indoors. Possibly still uses boot and cane for longer outdoor walks. Ankle likely at ~0° (neutral) or very close (maybe one small heel lift left). - Assess: FWB gait quality – can patient walk in boot with arms swinging naturally and no assistive device on level ground? Any pain at push-off (boot restricts motion but check discomfort)? Assess active plantarflexion strength out of boot (can patient press foot down against gravity or light resistance?). Measure ankle dorsiflexion with knee bent – may be at neutral or a few degrees shy. Calf circumference measure – compare to other side (will be smaller; document for progress). Review patient’s home exercise technique via video (ensure exercises are done with proper form). | - Goal: Wean off boot gradually and prepare for controlled shoe walking by ~6–8 weeks, while building foundational strength. - Mobilization: *Ankle:* If not at neutral, continue AROM until neutral is reached. Begin to allow mild dorsiflexion beyond neutral actively if no pain – but only a few degrees (do not force). *Weight-Bearing:* FWB in boot achieved; now start boot weaning: e.g. patient can remove boot for short periods at home or switch to a sneaker with a heel lift for 10–15 minutes at a time to acclimate (while seated or for very short supervised walks). Boot still used for longer walks and outside. - Sessions: Likely no full session this week unless issues – PT communicates via app messaging. If patient has difficulty with boot weaning, a quick video consult to demonstrate safe gait in shoes may be arranged. The timeline is individualized: ensure surgeon clearance before full discontinuation of boot. - Adjacent Joints: Add more functional leg exercises: e.g. step-ups on a 4–6” step (leading with injured side up and down) – this works knee and hip without excessive ankle motion. Also, gentle stationary lunge stance (split stance with injured leg back, heel stayed up) to stretch hip flexors and load calf lightly. Monitor that the back foot’s ankle doesn’t dorsiflex too far; use a small wedge under heel if needed. - Pain Mgmt: Pain is usually minimal; any moderate pain should be a red flag for overactivity. Advise continued icing after new exercises if mild swelling appears. By now, most will not need medication regularly. - Manual Therapy: Continue scar tissue and soft-tissue self-mobilization. If ankle joint feels stiff (patient cannot achieve neutral dorsiflexion easily), the therapist might instruct self-mobilization techniques (e.g. belt-assisted ankle dorsiflexion glide) – still very controlled. Mobilize adjacent joints if needed: e.g. gentle mobilization of subtalar joint by patient grasping hindfoot and moving it side to side. Encourage gentle self-massage on Achilles above and below repair site to improve tissue pliability (incision itself should be well healed by now). | - Strength & Balance: Seated single-leg calf raises: In sitting, lift heel of injured leg off floor (opposite foot stays down for support if needed); 3 sets of 10, 1×/day. Progress by adding a small weight on thigh for resistance. - Double-leg standing calf raise (assisted): With shoes on and hands on a counter for support, attempt to rise onto toes with both feet, sharing weight (injured leg will contribute modestly); do within pain-free range – maybe achieve only slight heel off ground; 2 sets of 10, 1–2×/day. - Theraband strength: Increase band resistance further for all ankle motions (PF, DF, inv, ever); 3×10 each, once daily. - Proprioception: Begin double-leg balance *without boot*: stand in stocking feet or shoes on flat surface, weight equally on both legs, and practice maintaining balance (hands hovering near support); try 30–60 sec, 3×/day. Also practice shifting weight toward injured leg while maintaining balance. - Cardio: Continue stationary bike (20+ min). If available, can introduce aquatic walking in shallow pool (chest-deep water) – walk forward and backward for 10 minutes (water reduces effective bodyweight ~50%, allowing gait practice with low impact). | - Adherence: Ensure patient follows the boot-weaning schedule strictly – e.g. if instructed to start wearing shoe with lift for 1 hour/day around the house, do not exceed that until confident. The app can include a weaning checklist (time out of boot each day, pain rating, etc.). Patient should continue diligent daily exercise; at ~5 weeks the Achilles will begin to tolerate more load, so this is a critical time to rebuild muscle. - Adjustments: If patient experiences increased swelling or soreness after trying shoes, return to boot and reduce activity for 1–2 days, then retry more gradually. Sleep in boot is typically still recommended until fully transitioned to shoes – if patient struggles with sleep, discuss with provider (sometimes a night splint at slight plantarflexion is allowed instead). Emphasize patience: even though daily function is improving, the tendon is still in early healing – avoid any impulsive high-force activities (running, jumping, lifting heavy loads on that leg). Encourage use of the app’s messaging or tele-chat if any uncertainty or if the patient feels the program is too easy/hard so it can be adjusted. |
| **Week 6** | - Status: ~6 weeks post-op. Milestone: Many patients can transition from boot to a supportive athletic shoe *with a heel lift* around this time. - Assess: Physician or PT evaluation this week to clear for boot discontinuation. Test pain-free ankle motion – dorsiflexion may now reach neutral or a few degrees beyond with knee bent (should not significantly exceed uninjured side’s resting tension). Check plantarflexion strength – patient should actively resist gravity (e.g. able to press foot down). Assess gait in normal shoes with a 1–1.5 cm heel insert: ensure they can perform a heel-to-toe gait without the boot (they may still use a cane briefly for confidence). Look at calf muscle activation during gait (should see some push-off effort, though weak). Confirm no tenderness along the tendon except post-exercise soreness. | - Goal: Wean off boot fully and normalize gait in shoes with heel lift; begin more dynamic strengthening. - Mobilization: *Ankle:* Gradually increase dorsiflexion range past neutral as tolerated (active motion). By end of week 6, patient can work toward dorsiflexing to ~5° (with knee bent) – still no forced stretching; let range improve through active use. *Weight-Bearing:* Full WB in shoes now. Start with heel lifts in shoes (to reduce tendon strain) and possibly an ankle brace for support. Initially, wear shoe with lift for a few hours at a time, intermix with boot if swelling or fatigue; by mid-week, aim for most of day in shoes. Gait training: focus on proper mechanics – encourage rolling onto toes gently (though push-off strength is limited). If needed, use one crutch or cane for balance during first days out of boot. - Sessions: In-depth tele-PT session at week 6 to guide gait training and exercise progression. PT may provide video demonstrations of advanced exercises (e.g. calf raises progression) via the app. This session also includes education: avoiding compensatory gait (like external rotation of foot), using mirrors or video to self-correct. Possibly schedule an in-person check with surgeon or PT this week for clearance tests. - Adjacent Joints: As walking in shoes resumes, monitor knee and hip alignment – continue strengthening hip abductors and core (to avoid Trendelenburg limp). Introduce gentle calf stretches for soleus with knee bent (e.g. seated ankle dorsiflexion) *if* patient feels very tight, but only mild stretch and not to pain (some protocols still avoid any deliberate stretch until 12 weeks, so this is optional and very gentle). - Pain Mgmt: Transition to heat therapy before exercise (to warm up stiff tendon) and ice after exercise if swelling. By now pain is mostly a “stretch” or soreness – use discomfort as a guide: a mild ache during exercise is okay, sharp pain is not. - Manual Therapy: Gait aid: Teach self-massage to plantar fascia and foot intrinsic muscles (since foot now bears weight, those areas might be stiff). Continue scar mobilization until scar is fully pliable. In lieu of therapist’s hands-on joint mobs, patient can use a slant board or rocker board gently to mobilize ankle (not stretching, just oscillatory movement into dorsiflexion). If available, gentle calf muscle mobilization (rolling pin or foam roller on calf) to ease muscle knots – avoid direct pressure on tendon repair site. | - Strengthening: Standing double-leg calf raises: Now perform with more weight on injured side as tolerated. Patient stands in shoes (with lift) and raises both heels off ground, focusing on equal weight distribution if possible; 3 sets of 10, 1×/day. Use a countertop for balance support. Eccentric control: Begin heel raise negatives: rise up on uninjured leg then shift weight to injured leg and slowly lower the heel down (eccentric loading); 2 sets of 5, 1×/day, if pain-free. (This helps tendon strength, but stop if painful or uncontrolled.) - Theraband & Toe Strength: Continue theraband in all directions (can increase to heavy band for PF). Add towel pickup drills: pick up marbles or a towel with toes to strengthen foot muscles (1–2×/day). - Balance/Prop: Single-leg stance (supported): Practice standing on injured leg (in shoe) for 10–15 seconds while lightly holding a counter for support; aim for 5–6 reps, 2×/day. Gradually reduce hand support to challenge balance. - Low-impact cardio: Increase stationary bike intensity or time (e.g. 20–30 min). If patient has access to an anti-gravity treadmill (AlterG) or pool, can initiate light jogging in reduced bodyweight environment (~25–50% BW) to start neuromuscular adaptation to running without full impact (optional, and only if guided by PT). | - Adherence: The patient is now taking more responsibility as formal supervision is less frequent. Emphasize consistency: daily exercises are crucial to regain strength (the calf will be very weak ~6 weeks post-op, needing regular stimulus to hypertrophy). The app can prompt the patient to log number of heel raises or single-leg balance time each day to show progress. Gait: Encourage patient to walk with full stride and confidence – it’s common to have a slight limp; using a mirror or video feedback can help. - Adjustments: If the patient cannot walk without a device by end of week 6 due to fear or weakness, plan an extra tele-session or even suggest an in-clinic visit for gait training. If any pain above 3–4/10 during weight-bearing, re-evaluate – might need to use boot a bit longer or check for issues (inadequate healing, too-rapid progression). Conversely, if patient is doing exceptionally well (no pain, normal gait), still caution against overtraining – the tendon is still remodeling. Maintain the heel lift in shoes until ~8 weeks even if feeling good, as it protects against sudden excessive stretch. |
| **Week 7** | - Status: 6–7 weeks post-op. The boot is typically discontinued fully by now; patient wearing normal shoes with a heel pad (~1 cm). Gait is FWB in shoes, possibly using a cane for uneven ground but generally walking independently indoors. - Assess: Quality of gait in different scenarios – level walking should be improving (maybe slight limp at push-off); assess gait on stairs: patient likely goes upstairs normally (using rail) but downstairs they might still go one step at a time (to avoid dorsiflexion strain). Check active dorsiflexion – likely a bit past neutral (~5–10° with knee bent). Plantarflexion strength – test a gentle bilateral heel raise: patient should be able to do a few reps of very small heel lift on both legs (injured side will produce minimal lift but should contribute). Measure single-leg balance time (can likely stand ~10–20 sec on injured leg). Inquire about any pain – mostly stiffness or soreness should be reported, not sharp pain. | - Goal: Normalize gait on level surfaces without assistive device, improve ankle ROM to functional range, and increase calf strength/endurance. - Mobilization: *Ankle:* Continue to increase active dorsiflexion range gradually. By end of week 7, aim for dorsiflexion ~10° (knee bent) if possible, but do not force it; let pain-free motion guide. May start very gentle passive stretch to Achilles if ROM is notably limited (e.g. low-load long-duration stretch with a towel, 5–10° into stretch, 30 sec) – only if no pain and with caution (many protocols still refrain from true passive stretches until week 12). *Weight-Bearing:* Full weight bearing in shoes across all daily activities. Practice uneven surface walking (e.g. on grass or carpet) to build confidence (with supportive sneakers). For stairs: introduce controlled eccentric step-downs off a low step (holding rail) to begin training for going downstairs normally – only if Achilles can control it without pain. - Sessions: Likely no formal session this week; continue remote monitoring. The PT might send new exercise videos through the app (progressions for strengthening, mild plyometrics in water, etc.). If patient had any setbacks in transitioning out of boot, a quick video consult ensures they are on track now. - Adjacent Joints: Increase focus on whole-leg function: add closed-chain knee exercises like partial lunges (only go to where injured ankle allows). Continue hip strengthening (e.g. single-leg stands with hip abduction of opposite leg to simulate gait stability). Ensure opposite limb is staying strong as well (to avoid asymmetry). - Pain Mgmt: Usually just stiffness now. Suggest warm showers or heat before exercise to loosen Achilles. If swelling occurs after long walks, use ice and elevate. Educate about soreness vs. pain: mild calf soreness after exercise is normal; sharp pain or swelling that lasts into next day means to scale back. - Manual Therapy: At this stage, scar adhesions should be resolved; if not, continue aggressive massage. *Ankle joint mobs:* if dorsiflexion is limited, the patient can perform self joint-mobilization by lunging gently with foot on a wedge (to bias talus glide) – or the PT can guide a spouse/partner to help gently mobilize the ankle if possible. Soft tissue: continue foam rolling calf and even gentle cross-friction massage to tendon *if* advised (to help remodeling, but only very gently along the tendon, not across it, and stop if any irritation). | - Strength: Bilateral heel raises (in shoes): Continue daily; by now aim for improved height. Do 3 sets of 15 reps, focusing on slow control, 1×/day. After each set, stretch calves lightly (no bouncing). - Single-leg heel raise (assisted): Begin training injured leg by itself: stand on injured leg, use fingertips on wall for balance and assist by off-loading weight (e.g. lightly push with opposite foot or arms) to attempt a calf raise; even if only a few centimeters of lift. Do 2–3 sets of as many reps as can be done with good form (quality over quantity), 1×/day. It’s expected the patient may not achieve full single-leg raises yet – this is practice to activate neuromuscular control. *Note:* Only ~50% of patients can do a single-leg heel raise at 12 weeks, so encourage effort but manage expectations. - Theraband/BFR: To further hypertrophy, consider blood-flow restriction (BFR) training if patient has access to a cuff and guidance: low-load resistance (e.g. long Theraband or bodyweight calf raises) with BFR can significantly boost strength gains. If unsupervised BFR is not available, continue increasing resistance with traditional exercises (e.g. add a backpack for more weight during heel raises). - Balance/agility: Single-leg balance: aim for 30+ seconds unaided. Add perturbations (e.g. toss a ball against wall while standing on injured leg) to increase challenge. - Dynamic drills: Start gentle side-to-side weight shifts and heel-to-toe rocking in place to prepare for faster movements. If in pool, can do gentle bounding or skipping in chest-deep water. If on land, begin using a trampoline or soft surface for mini-bounces (keeping toes on ground, not full jumping). - Cardio: May introduce elliptical machine or stair-climber towards end of this week if gait is normalized (criteria: patient can go up an 8” step with good form); start low resistance, short duration. | - Adherence: By week 7, the novelty of rehab may wear off – motivate the patient to stay consistent. Use the app’s progress charts (strength gains, balance time, etc.) to show improvements, reinforcing that exercises are working. Encourage integration of rehab into daily routine (e.g. do calf raises every time waiting for the microwave, balance while brushing teeth, etc.). - Adjustments: Increase or decrease exercise volume based on soreness: employ a “soreness rules” approach – if muscle soreness from yesterday’s exercise is > 2/10 next day, do a lighter day; if no soreness, can gradually increase reps or resistance. If any movement (like elliptical) causes pain or asymmetrical gait, hold off another week before retrying. Continue to wear the heel lift if instructed (usually until ~8 weeks); removing it too early can overstretch the tendon. Patient should also continue avoiding barefoot walking – wear supportive shoes even at home, as barefoot can put sudden stretch on Achilles. |
| **Week 8** | - Status: 7–8 weeks post-op. Milestone: Likely okay to remove heel lifts around end of week 8 if dorsiflexion is adequate and gait is good. Patient walking in normal shoes now most of the time. - Assess: Formal re-evaluation around 8 weeks: measure ROM (goal ~0° dorsiflexion knee straight, ~10–15° with knee bent by now), calf strength (how many bilateral heel raises, quality of movement), single-leg heel raise attempt (probably still partial or needing support), and functional tests like a light jog in place or gentle hop in pool (if applicable). Gait should be near-normal on level ground; assess any deviations (e.g. lack of push-off or slight limp). Evaluate psychological readiness – does patient feel confident in the tendon? This can affect performance of exercises. | - Goal: Full transition to normal footwear without heel lifts, near-normal gait and begin early plyometric and jogging prep (if appropriate). - Mobilization: *Ankle:* By week 8, gentle passive stretching of the Achilles can be initiated if needed: e.g. standing calf stretch against wall – go only until a mild stretch is felt, hold 20–30 sec, 3×. Continue increasing dorsiflexion as strength allows; should be approaching needed range for stairs (about 15°). *Weight-Bearing:* All regular daily activities in shoes. Practice stair descent with proper form: encourage eccentric control (slow lowering) using railing support. If not yet doing so, train patient to go downstairs foot-over-foot as strength permits (still okay to go one step at a time if unsure). - Sessions: Telehealth or in-person check at 8 weeks to progress program. PT may do a movement assessment: check for compensations like toe-out gait or knee hyperextension. Based on findings, refine exercises (e.g. add specific drills if patient avoids push-off or lacks ankle mobility). Discuss timeline for returning to higher impact – emphasize they are entering a “late rehab” phase focused on rebuilding power and endurance, not there yet for full sports. - Adjacent Joints: If any residual deficits (e.g. slight knee stiffness or hip weakness from initial immobility), address with targeted exercises (like deeper knee bends, hip strengthening with resistance). Incorporate more whole-body functional moves: light squat-to-calf raise combinations, gentle skipping motions, etc., to integrate the kinetic chain. - Pain Mgmt: Pain should be negligible at rest. Muscle soreness after more intense exercise is expected. Manage with active recovery (e.g. easy cycling or swimming) and stretching. If tendon itself is sore, back off intensity and use ice and compression. - Manual Therapy: Now that the tendon is more robust (~8 weeks, fibroplasia phase transitioning to remodeling), more assertive manual techniques can be used if needed. E.g. deep transverse friction massage to the Achilles if there are thickened fibrotic areas (done carefully, perhaps taught for patient to do mild version, or done in clinic). Continue joint mobilizations to ensure talocrural and subtalar joints have full mobility (especially if dorsiflexion still limited – posterior talar glide techniques). Soft tissue work on calf and even plantar fascia (since gait changes can cause foot tightness) is beneficial. | - Advanced Strength: Single-leg calf raises (eccentric focus): If patient still cannot lift fully, emphasize eccentrics: do 3 sets of 10 slow eccentrics (rise on both feet, lower on injured leg). If already can do a bit of single-leg, practice at a counter: aim for 1–3 unassisted single-leg heel raises with good form by end of week 8 (even partial range). - Double-leg plyometrics: Introduce light bouncing drills on both legs: e.g. two-legged ankle hops in place (feet together, do small hops focusing on quick rebound and using calf); start with 2×10 hops, 1–2×/day. (Ensure patient is wearing supportive shoes and floor is not slippery; these simulate the stretch-shortening cycle gently.) - Functional exercises: Lunges: Forward lunges or split squats, keeping injured leg behind to limit DF, 2×10 each side. Step-ups: Increase step height to challenge (6–8” step), drive up with injured leg; 2×10. Heel-to-toe walking: Slow exaggerated gait on tip-toes for several steps, then on heels (if able), to improve calf and anterior tib strength – ensure support nearby. - Balance: Progress to unstable surface (e.g. foam pad or pillow) single-leg standing, 3×30 sec. Add dynamic balance: single-leg mini squat (bend knee slightly and straighten). - Endurance: If cleared by physician, initiate a graded jogging program on a treadmill or track by end of week 8 or early week 9: e.g. begin with brisk walking, then light jog for 1 minute, walk 2 minutes, for a total of 10–15 minutes. Keep intensity low and focus on form (no pushing off too hard). Stop if pain > 2/10. Many protocols start running around 12 weeks, but some athletes may begin light jogging at 8–10 weeks if strength is sufficient and under guidance. This must be individualized. | - Adherence: As activities diversify (strength, balance, light running), the patient must balance volume and rest. Ensure they use the app’s scheduling feature to space out high-load days with easier recovery days. Continue to fill out outcome measures in app (e.g. Achilles Rupture Score) to track improvement in function and symptoms. - Adjustments: If swelling or stiffness spikes with introduction of jogging or plyometrics, advise a 1–2 day rest and regress to gentler exercises before trying again. Communication remains key: patient should report any pain in the tendon (not just muscle soreness) that lasts >24 hours, as this could indicate overloading. The program may be adjusted to slower progression if needed. Conversely, if the patient finds the exercises too easy and has met all milestones (e.g. can do 10 single-leg heel raises, full ROM), the PT can advance difficulty (heavier resistance, more challenging balance) even faster. However, remind that tendon integrity is the rate-limiter – it remodels gradually, so even if symptoms are gone, they should avoid maximal calf efforts or return to explosive sports until later (~3–6 months). |
| **Weeks 9–11** | - Status: 8–11 weeks post-op. During this interval, patient is improving strength and endurance. By week 10, they often achieve full ankle ROM (or very close) and can do at least a few assisted single-leg heel raises. Gait on level ground is normal or with minimal deficiency (perhaps slight weakness in push-off). They can likely jog lightly (if initiated) for short durations by week 10–11. - Assess (ongoing): At week 10 or 12, a re-assessment by PT or surgeon may be done: check single-leg heel raise height (expect still < full height of uninjured side), number of continuous bilateral calf raises (should be high, e.g. 20+ reps), hopping ability (maybe beginning to hop in place on injured leg by week 11 if cleared), and any residual swelling. Test functional tasks: e.g. can patient do a quick direction change or cut at slow speed without pain? (Probably not fully until >12 weeks, but can trial at slow speed to see confidence.) | - Goal: Progress towards full unilateral calf strength and prepare for return-to-run and plyometric phase after 12 weeks. Address any remaining deficits (strength, balance, flexibility) proactively. - Mobilization: *Ankle:* By week 10, begin dynamic stretching as appropriate (e.g. calf stretch with knee straight to target gastrocnemius, since knee-bent dorsiflexion likely restored). Continue to avoid any aggressive ballistic stretches. *Weight-Bearing:* Add more challenging weight-bearing tasks – e.g. light agility ladder drills (hopping or running through ladder with small steps) if patient is jogging. In daily life, patient should be able to walk on various terrains, carry groceries, go up/down stairs normally by end of week 11 (downstairs may still be slower than normal). - Sessions: Possibly a check-in around week 10 to refine advanced exercises. If patient is an athlete, discuss sport-specific goals and introduce pertinent drills (within safety limits). E.g. for a soccer player, start simple ball dribbling drills while walking; for a runner, focus on form and cadence during short jogs. Ensure patient has a plan for post-12-week training (they may continue formal rehab or transition to independent gym program). - Adjacent Joints: At this stage, include plyometric conditioning for other joints: small hops and jumps not just using ankle but also knees/hips (like mini squat jumps) to gradually reintroduce impact to the whole limb. This helps the knee and hip absorb load when full return to sport begins, distributing stress away from the healing Achilles. - Pain Mgmt: By now, pain is mostly absent; occasional morning stiffness in Achilles is normal. Warm-up routines in the morning (ankle circles, light stretching) can help. If a particular exercise causes lasting pain, scale it back and use symptomatic treatment (ice, NSAIDs sparingly) and inform PT. - Manual Therapy: Any final manual interventions to address limitations: e.g. if ankle dorsiflexion is still < 90% of other side, more aggressive joint mobilization (grade III-IV posterior glide of talus) could be done by a PT in clinic. Deep tissue massage to calf and tendon can be done to ensure optimal scar remodeling (patient can do some at home with massage tools). Focus on scar desensitization if scar is hypersensitive (rub with different textures). Overall, manual therapy now is targeted as needed, not routine. | - Advanced Exercises: Single-leg heel raises: By week 10–12, attempt unassisted single-leg calf raises for reps. Goal is to perform ~5–10 by week 12, though form and height may not be perfect. Keep doing eccentrics and bilateral raises to build toward this. - Jump and hop training: Increase intensity: e.g. Single-leg hops in pool or on trampoline by week 10 (if tolerated). By week 11, try double-leg jump rope or gentle jogging in place on land. Introduce bounding drills (longer steps or skips) to improve power, if patient can jog. Always emphasize soft landings and proper form (avoid pushing through pain). - Strength training: Incorporate more resistance training: leg press calf raises (if gym available), gradually increasing resistance but high reps (15–20) to build endurance. Include eccentric heel drops off a step (drop heel below step level slowly) by week 11 if flexibility allows – this can help lengthen the muscle-tendon unit safely and increase strength at end-range. Continue other leg strengthening (squats, lunges can be deeper now, step-ups can be faster or higher step) to ensure the entire kinetic chain is strong. - Agility: Begin gentle cutting or lateral movements: e.g. side-stepping drills, grapevine/carioca steps at slow speed to challenge coordination. Use cones to practice changing direction gradually. - Endurance: Extend jogging intervals (if started). By week 12, patient might jog continuously for 10+ minutes if no issues. If they are not jogging yet, focus on brisk walking and try fast walking up an incline to build strength in a similar way. | - Adherence: At this point, the patient may feel much closer to normal and be tempted to skip the “boring” exercises. Remind them that full strength recovery in the calf is a longer process – structured exercise should continue for many months. Encourage using the app’s goal-setting feature to set new targets (e.g. jog 1 mile, do a single-leg heel raise with full height). The app can also prompt functional fitness tasks (like “today, try descending stairs without holding the rail”). - Adjustments: Tailor the plan to patient’s goals – if they only need to return to recreational activities, focus on those specific tasks; if they are high-level athletes, a formal return-to-sport testing will be planned typically after 3–6 months, but the groundwork is laid now. Ensure the patient doesn’t attempt return to competitive play or maximal efforts yet – discuss any plans with their provider. For any plateau in strength (e.g. struggling with single-leg raises), consider adding modalities like neuromuscular electrical stimulation (NMES) during exercises to improve muscle recruitment, or increase supervision frequency briefly to push past the plateau. Continual feedback and slight program tweaks (via telehealth or messaging) keep the patient engaged and progressing. |
| **Week 12** | - Status: 11–12 weeks post-op. Milestone: End of early rehab phase. Patient should have nearly full ROM in the ankle (maybe a slight loss in end-range dorsiflexion compared to uninjured side, which often normalizes later). They walk normally at various speeds and can go up/down stairs reciprocally, though may still do so carefully. They can do some heel raises (likely still working on single-leg strength). Impact activities are in the beginning stages (light jogging, small hops). - Assess: A comprehensive evaluation is done. Key assessments: Achilles Tendon Total Rupture Score (ATRS) or similar patient-reported outcome – expecting significant improvement from initial scores. Strength tests: single-leg heel raise count and quality, isokinetic testing if available (to quantify plantarflexion torque deficit – likely still 30–50% deficit at 3 months). Functional tests: e.g. double-leg hop for distance (compare sides), single-leg balance reach tests, perhaps a gentle single-leg hop test if appropriate (though 90% hop symmetry is usually not until ~6 months). Ensure psychological readiness – patient should feel confident in basic movements. Discuss follow-up plan (further PT or independent program). | - Goal: Discharge criteria for early rehab: safe independent function in daily activities, and a plan for ongoing strengthening. By 12 weeks the protocol aims for: Ankle dorsiflexion ~90–100% of other side; ability to do at least 1–2 single-leg calf raises (even if not full height); normalized walking and basic jogging mechanics; no pain with ADLs. The goal now is to transition to more advanced training for return to full sport by ~6 months. - Mobilization: *Ankle:* Full end-range mobilization is now allowed – include weight-bearing stretches (both gastrocnemius and soleus stretches) and joint mobs as needed to eke out final degrees of motion. *Weight-Bearing:* Begin higher impact loading carefully: e.g. start a formal running progression if not already – gradually increase jogging duration and speed over next few weeks. Also initiate plyometric progression: start from double-leg jumps to more single-leg hops in controlled settings. Emphasize quality over quantity to avoid bad habits. Sessions: Final telehealth session for this phase to review progression and ensure patient understands next steps. PT provides a detailed maintenance program to follow beyond 12 weeks, possibly including gym strengthening, agility drills, and stretching routine. They also discuss criteria for return to sports and the importance of not rushing (will likely need clearance tests around 4–6 months). - Adjacent Joints: At discharge, ensure no secondary issues: sometimes patients develop hip or knee soreness from altered gait – address with appropriate exercises or refer for continued therapy if needed. Advise on whole-body conditioning to regain any general fitness lost during early rehab (e.g. gradually reintroduce running, jumping, change of direction drills that involve all joints). - Pain Mgmt: At 3 months, the tendon is much stronger but still remodeling. Some morning stiffness or mild ache after a heavy day’s activity can persist – patient should manage this with self-care: gentle stretches, ice if swollen, or contrast baths. They should monitor the tendon for any signs of regression (e.g. increasing pain or swelling) and moderate their activities accordingly. - Manual Therapy: Encourage the patient to continue self-massage and mobilization techniques as needed, even beyond formal rehab. Regular foam rolling of the calf, ankle mobility drills, and occasional self-massage to the tendon can help maintain flexibility and tissue quality. If significant tightness or scar tissue concerns remain, advise periodic check-ins or a session of instrument-assisted soft tissue mobilization (ASTM) or similar in a clinic. | - Performance & Strength: By week 12, incorporate more sports-specific or functional drills relevant to the patient’s goals. For example: start light jump shots for a basketball player (focus on form, minimal jump); for a runner, introduce tempo runs or gentle hills (if strength permits); for a dancer, practice rises and small hops. All new activities should be introduced at low intensity and volume. - Strength training: Continue progressive overload – e.g. single-leg calf raises every other day aiming to slowly increase reps. Add plyometric exercises like box jumps (small box) or lateral jumps over a line, if appropriate, with emphasis on soft landing and no knee collapse. By now the patient can also start resisted gait drills (like pulling a sled or resistance band around waist for short marches) to build power. Neuro-muscular training: Include drills for agility and reaction (e.g. shuttle runs, ladder drills) at a low intensity to redevelop coordination. Balance training can advance to plyometric level: e.g. hop-to-balance (hop forward on injured leg and stick the landing). - Endurance: Patient should work up to jogging continuously 15–20 minutes if returning to running sports. For others, ensure they can tolerate equivalent cardio (e.g. 30 minutes cycling or elliptical without issues). They can also start swimming (flutter kick is usually safe now as ankle motion is restored). - Home program beyond 12 weeks: Emphasize the continuation of strengthening and gradual return to high-impact. Provide a written or app-based extended program (weeks 12–24+) that includes progression of running (intervals to continuous), jumping (small hops to higher jumps), and sport drills, along with maintenance stretching and strengthening. | - Adherence: Transitioning out of supervised rehab can be tricky – stress that adherence to the independent program will determine the success of full recovery. The patient should set long-term goals (e.g. “in 3 months, jump and grab the rim again” or “return to full tennis by 6 months”) and use those to stay motivated. The app can continue to be used in self-guided mode, sending reminders and allowing the patient to log progress even without regular PT oversight. - Adjustments: Ensure the patient knows to listen to their body: even at 12 weeks, doing too much too soon (like sprinting or playing a pickup game) can risk re-injury. They should increase intensity in increments and ideally not return to full competition until cleared by a physician/therapist with objective tests (usually at 4–6 months when limb symmetry index >90% is achieved in strength and hop tests). If any new issues arise (e.g. tendon pain or other joint pain as they ramp up training), they should seek a follow-up with PT or doctor. Finally, celebrate progress – the patient has come a long way in 3 months. Continued dedication will yield a return to full activities on schedule, as studies show accelerated rehab yields high patient satisfaction and excellent functional outcomes at final follow-up. |

NWB: none-weight bearing; WB: weight bearing; ROM: range of motion; AROM: active range of motion; DVT: deep vein thrombosis; ASTM: instrument-assisted soft tissue mobilization; ATRS: Achilles Tendon Total Rupture Score; PT: physiotherapist.

In DEP group, patients largely self-manage exercises at home using a rehabilitation app with instructional videos and reminders. A physical therapist provides telerehabilitation check-ins (video or phone) at critical points (around weeks 2, 4, 6, 8, 12) to assess progress, adjust the program, and ensure safety. The patient logs daily exercises, pain levels, and any issues in the app, enabling remote monitoring. Early weight-bearing in a protective boot and early active motion are utilized to accelerate recovery while safeguarding the repair

In CP group, patients attend regular physiotherapy sessions (typically 3 times per week) at participating hospitals and two other assigned PT clinics in Shanghai. The therapist provides hands-on treatments and closely supervises exercises and gait training. This in-person guidance can reassure the patient and allow for immediate technique corrections. The clinic-based protocol follows the same timeline of milestones as the home-based program. Notably, early weight-bearing and mobilization are still emphasized – the physiotherapist works in tandem with the surgeon to implement an accelerated rehab safely

**References**

1. Brumann M, Baumbach SF, Mutschler W, Polzer H. Accelerated rehabilitation following Achilles tendon repair after acute rupture - Development of an evidence-based treatment protocol. Injury. 2014;45(11):1782-90.
2. Zhao JG, Meng XH, Liu L, Zeng XT, Kan SL. Early functional rehabilitation versus traditional immobilization for surgical Achilles tendon repair after acute rupture: a systematic review of overlapping meta-analyses. Sci Rep. 2017;7:39871
3. Massen FK, Shoap S, Vosseller JT, et al. Rehabilitation following operative treatment of acute Achilles tendon ruptures: a systematic review and meta-analysis. EFORT Open Rev. 2022;7(10):680-691.
4. Nilsson-Helander K, Silbernagel KG, Thomeé R, et al. Acute Achilles tendon rupture: a randomized, controlled study comparing surgical and nonsurgical treatments using validated outcome measures. Am J Sports Med. 2010;38(11):2186-93.
5. Kangas J, Pajala A, Ohtonen P, Leppilahti J. Achilles tendon elongation after rupture repair: a randomized comparison of 2 postoperative regimens. Am J Sports Med. 2007;35(1):59-64.
6. Okoroha KR, Ussef N, Jildeh TR, et al. Comparison of tendon lengthening with traditional versus accelerated rehabilitation after Achilles tendon repair: a prospective randomized controlled trial. Am J Sports Med. 2020;48(7):1720-1726
7. Aufwerber S, Heijne A, Edman G, Silbernagel KG, Ackermann PW. Does early functional mobilization affect long-term outcomes after an Achilles tendon rupture? A randomized clinical trial. Orthop J Sports Med. 2020;8(3):2325967120906522.
8. Valkering KP, Aufwerber S, Ranuccio F, Lunini E, Edman G, Ackermann PW. Functional weight-bearing mobilization after Achilles tendon rupture enhances early healing response: a randomized controlled trial. Knee Surg Sports Traumatol Arthrosc. 2017;25(6):1807-1816.
9. Hansen OB, Papson A, Eble SK, Drakos MC. Effect of blood flow restriction therapy following Achilles rupture and repair: a randomized controlled trial. Foot Ankle Orthop. 2022;7(1):1-7 (eCollection)
